# Protective effect of 6-paradol in acetic acid-induced ulcerative colitis in rats

**DOI:** 10.1186/s12906-021-03203-7

**Published:** 2021-01-13

**Authors:** Misbahuddin Rafeeq, Hussam Aly Sayed Murad, Hossam Mohammed Abdallah, Ali M. El-Halawany

**Affiliations:** 1 Department of Pharmacology, Faculty of Medicine, King Abdulaziz University (KAU), Rabigh Campus, Jeddah, 21589 Saudi Arabia; 2 Department of Pharmacology, Faculty of Medicine, Ain Shams University, Cairo, 11562 Egypt; 3 Department of Natural Products and Alternative Medicine, Faculty of Pharmacy, KAU, Jeddah, 21589 Saudi Arabia; 4 Department of Pharmacognosy, Faculty of Pharmacy, Cairo University, Cairo, 11562 Egypt

**Keywords:** 6-paradol, Spices, Ginger, Grain of paradise, Gut, IBD, Antioxidant

## Abstract

**Background:**

Ulcerative colitis is a gut inflammatory disorder due to altered immune response to gut microbiome, with interplay of environmental and genetic factors. TNF-α activates inflammatory response through a cascade of immune responses, augmenting pro-inflammatory mediators and proteases, activating chemotaxis, and infiltration of inflammatory cells, leading to ulceration and haemorrhage through cytotoxic reactive oxygen species. 6-Paradol, a dietary component in several plants belonging to the Zingiberaceae family, has shown anti-inflammatory and antioxidant activities. Current study evaluates the effect of 6-paradol in amelioration of ulcerative colitis in rats for the first time.

**Methods:**

6-Paradol (95% purity) was obtained from seeds of *Aframomum melegueta.* Rats were divided randomly into six groups (*n* = 8). Group one was administered normal saline; group two was treated with the vehicle only; group three, sulfasalazine 500 mg/kg; and groups four, five, and six, were given 6-paradol (50, 100, 200, respectively) mg/kg orally through gastric gavage for 7 days. Colitis was induced on 4th day by intrarectal administration of 2 ml acetic acid (3%), approximately 3 cm from anal verge. On 8th day, rats were sacrificed, and distal one-third of the colon extending proximally up to 4 cm from anal orifice was taken for biochemical and gross examination. Two centimetres of injured mucosal portion was taken for histopathological investigations. SPSS (ver.26) was used for statistical analysis.

**Results:**

Colonic and serum glutathione (GSH) levels decreased, while colonic and serum malondialdehyde (MDA), colonic myeloperoxidase (MPO) activity, serum interleukin-6 (IL-6), serum tumour necrosis factor-α (TNF-α) levels, and colon weight to length ratio were increased significantly in the colitis untreated group compared to normal control. Treatment with 6-paradol considerably improved all these parameters, especially at a dose of 200 mg/kg (*p* < 0.001), revealing non-significant differences with sulfasalazine 500 mg/kg and normal control (*p* = 0.998). Sulfasalazine and 6-paradol in a dose dependent manner also markedly reversed mucosal oedema, atrophy and inflammation, cryptic damage, haemorrhage, and ulceration. There were non-significant differences between low and medium doses and between medium and high doses of 6-paradol for IL-6 and serum MDA levels.

**Conclusion:**

6-Paradol demonstrated protection against acetic acid-induced ulcerative colitis, probably by anti-inflammatory and antioxidant actions.

**Supplementary Information:**

The online version contains supplementary material available at 10.1186/s12906-021-03203-7.

## Background

Inflammatory bowel disease (IBD) is an idiopathic, chronic, and relapsing gut inflammatory disorder speculated to be caused by an altered immune response to the gut microbiome. In addition, environmental and genetic factors also play roles in its etiopathogenesis [1]. Two major types of IBD include Crohn’s disease (CD) and ulcerative colitis (UC). In UC, there is localized superficial inflammation limited to the colon and rectum, while in CD, the whole intestine is involved with ulceration, haemorrhage and oedema. Moreover, the involvement is transmural, meaning that all layers of the intestinal wall are involved [2].

Tumour necrosis factor-α (TNF-α) along with IL-2 and INF-γ, are from T-helper1 (Th1) cells. A preponderance of these factors over mediators from T-helper2 (Th2) cells, such as IL-4, IL-5, and IL-6, results in the development and progression of allergic and autoimmune disease [3]. TNF-α plays a prudent role in the development of UC by augmenting the inflammatory response through activation of a cascade of immune responses [4]. TNF-α also activates the production of other chemical mediators, proteases, and pro-inflammatory markers, activating chemotaxis and infiltration of inflammatory cells, leading to ulceration and haemorrhage, by generation of cytotoxic reactive oxygen species [5, 6].

The main symptoms include abdominal pain with cramping, diarrhoea, dysentery, constipation, fatigue and fever. The diseases are important causes of increased healthcare expenditure and socioeconomic burden, as the Quality Adjusted Life Years (QALYs) increase due to frequent relapses and exacerbations. The highest prevalence of IBD is found in the US, Canada and Western Europe, with over 1 million in the US and 2.5 million in Europe. Nevertheless, increasing numbers of patients are also being diagnosed in the Middle East and Saudi Arabia [7, 8]. The therapy revolves around anti-inflammatory and immune-modulatory drugs, such as sulfasalazine, corticosteroids, methotrexate, and infliximab (anti-TNF-α). Often, antibiotics and probiotics are also used for infections and abscess, healing of fistulas, and resetting of the gut microbiome [9, 10].

Natural products have long been used for alleviating diseases and are considered as the major source of most of the discovered medicines [11, 12]. Consequently, searching for evidence for the use of herbal medicines (especially those with historic use in the treatment of GI disorders) in the treatment of IBD is worthy and beneficial. 6-Paradol is a major phenolic dietary component that occurs in several plants belonging to the Zingiberaceae family, such as *Zingiber officinale* (ginger) and *Aframomum melegueta* (alligator pepper or grains of paradise). The plants of this family have been evaluated for their possible role in the prevention and treatment of gut inflammatory disorders [13, 14]. Anti-inflammatory, antioxidant, gastroprotective, immunomodulatory, antinociceptive, antimicrobial, antidiabetic, and anticancer activities have been reported from the extract of *A. melegueta* and its active components [15–17]. In addition, previous reports also advocate anticancer, anti-diabetic, antioxidant, anti-inflammatory, and immunomodulatory activities of 6-paradol, which is one of the major constituents of grains of paradise [18–20]. Effects of other individual active constituents from the Zingiberaceae family, such as such as gingerols, shogaols and gingerdione, on ulcerative colitis and other inflammatory models have been investigated; however, no study has been conducted with paradol. Hence, the present study was designed to explore the protective effect of 6-paradol in acetic acid-induced ulcerative colitis in rats.

## Methods

### Plant material

*A. melegueta* seeds were procured from Harraz herbal store, Cairo, Egypt, in 2018, and were identified by Dr. Sherif El-Khanagry, Agriculture Museum, Dokki, Cairo. A specimen (voucher ID: AM-1039) was deposited in the herbarium of the Department of Pharmacognosy, Faculty of Pharmacy, King Abdulaziz University.

### Extraction and isolation of 6-paradol from the seeds of *Aframomum melegueta*

6-Paradol was isolated as described briefly in our previous report; pulverized seeds of *A. melegueta* (1.0 kg) were extracted using methanol by maceration at room temperature until exhaustion. The combined methanol extracts were evaporated under vacuum, yielding an oily brownish residue (TME, 50 g). TME was suspended in water (300 mL), followed by partitioning with methylene chloride (500 mL X 3) to yield, after evaporation, a CH_2_Cl_2_-soluble fraction (30 g). The remaining aqueous layer was freeze-dried and kept for further investigations. The CH_2_Cl_2_ fraction was applied to a silica gel column (50 X 4 cm) and gradient-eluted using n-hexane-EtOAc (5 until 80% v/v). One hundred millilitre fractions were collected and pooled based on TLC investigation into 10 sub-fractions (F1-F10). F1 (6 g) was chromatographed on a silica gel column (40 X 4 cm) eluted with hexane-EtOAc (9.5:0.5 v/v) to obtain pure paradol (5 g). The identity of the isolated compound was confirmed by 1H and 13C NMR analysis recorded on Bruker DRX-850 MHz Ultrashield spectrometers (Bruker BioSpin, Billerica, MA, USA) using CDCl_3_ as solvent, with TMS as the internal reference. The purity of isolated 6-paradol was 95%.

### Animals and drugs

Male Sprague-Dawley rats, aged 10–12 weeks, with a weight range of 250–300 g were procured from King Fahd Medical Research Center. They were handled and housed under standard laboratory conditions in polypropylene cages with not more than six rats per cage under room temperature (25 ± 2 °C) with a 12-h light-dark cycle and given standard chow with water ad libitum during the entire experimentation phase. Drugs and chemicals were acquired from Sigma-Aldrich Corp. (St. Louis, MO, USA) unless stated elsewise. The study protocol was approved by the King Abdulaziz University Research Ethics Committee (KAU-REC), no. E-157/38, and all animal experimentation was performed in accordance with the International Guidelines for the Care and Use of Laboratory animals [21], and ethical code according to the National Committee of BioEthics (NCBE), King Abdulaziz City for Science and Technology, Saudi Arabia.

### Experimental design

- Treatment groups
- Induction of colitis

A total of 48 animals were randomly divided into six groups as follows, with each group consisting of eight animals (*n* = 8). Group 1: normal control group (NC) given normal saline; 2: acetic acid-induced colitis untreated group (AC) with vehicle only; 3: treatment group with sulfasalazine 500 mg/kg (SG); 4: treatment group with 6-paradol 50 mg/kg (LT); 5: treatment group with 6-paradol 100 mg/kg (MT); 6: treatment group with 6-paradol 200 mg/kg (HT).

All the above groups were given the stated treatments from 0 to 7 days through gastric gavage once in the morning. Under light sedation with ether, colitis was induced on the 4th day by intra-rectal administration of 2 ml of acetic acid (3% w/v) through a lubricated catheter. The instillation site was approximately 6–8 cm from the anal verge. In rats, colitis induced by acetic acid depicts a comparable pathology to IBD in humans, which consists of an increase in inflammatory mediators, localized involvement and damage to the intestinal epithelium [22]. On day 8, blood was withdrawn, and serum was kept at − 80 °C for estimation of levels of glutathione (GSH), malondialdehyde (MDA), TNF-α, and IL-6. Then, the rats were euthanized by cervical dislocation, and the colon samples were collected for analysis of myeloperoxidase (MPO) activity and measurement of GSH and MDA levels, in addition to macroscopic study and histopathological examination.

### Biochemical determination of colon inflammation

#### Colonic myeloperoxidase (MPO)

Colonic myeloperoxidase (MPO) was measured by the method described by Lefkowitz et al. 1992 [23]. Scrapings weighing approximately 100 mg from the colonic mucosa were homogenized in 0.5% HTA bromide and then dissolved in KPO_4_ buffer (50 mM, pH 6) and sonicated for 10 s in an ice bath. This homogenate mixture was then frozen and thawed thrice and placed in a centrifuge (20,000 rpm, 15 min). Afterwards, 2.9 ml of KPO_4_ buffer (50 mM) containing additional H_2_O_2_ and O-dianisidine dihydrochloride was mixed, and 0.1 ml of the supernatant was taken. The absorbance of this solution was read for 5 minutes at 460 nm by means of a spectrophotometer (Beckman DU 640B).

#### Colonic glutathione (GSH)

Colonic glutathione (GSH) levels were measured by an assay kit (Cayman Chemical Company, US). After preparation of homogenate from colonic mucosa, it was centrifuged after being mixed with an equal amount of TCA (20%). Then, 0.25 ml of this supernatant was taken and mixed with 0.75 ml of KPO_4_ buffer (pH 8.0). This solution was further supplemented with 2 ml of Ellman’s reagent. It was then analysed at 412 nm by spectrophotometer against reagent blank [24].

#### Colonic malondialdehyde (MDA)

Colonic malondialdehyde (MDA) was assessed based on the method described by Ohkawa et al., 1979 [25]. After taking 2 ml of homogenate from colonic mucosa, it was mixed with an equal amount of TCA (10%w/v) and centrifuged at 20,000 rpm after freezing for 15 min. Afterwards, 2 ml of TCA was again mixed with 2 ml of the supernatant. The solution was heated for 10 min at 100 °C and rapidly cooled at 0 °C for 5 min. The concentration was read at a wavelength of 535 nm.

### Determination of inflammatory markers and antioxidants in serum

Blood collection was performed in clot activator tubes (DD Biolab SL, Barcelona, Spain), and serum was separated by centrifugation (4 °C) at 3000 rpm. IL-6 and TNF-α levels were measured using commercially available ELISA kits (Gentaur molecular products, USA). Serum GSH and MDA measurements were performed using colorimetric kits (Cell Biolabs, San Diego, CA, USA) as per the manufacturer’s protocol.

### Macroscopic assessment of colonic damage

Macroscopically visible colonic lesions such as mucosal oedema, thickening, bleeding, hyperaemia, erosions, shortening, and necrosis were assessed. The lesions were classified as normal, mild, moderate, and severe [26]. The weight by length ratio of the colon was also determined by measuring weight in a balance and assessing length by scale after longitudinally opening and rinsing the colon gently under water.

### Histopathological examination

Colonic specimens were fixed in phosphate-buffered formalin (10%), embedded in blocks of paraffin, and sliced into 3- to 5-μm sections, stained with H&E and were assessed for mucosal damage, ulceration, erosions, haemorrhage, and necrosis by a pathologist in blinded manner under light microscopy. The severity of the lesions was annotated as either normal, mild, moderate or severe [27]. These methods were utilized by our team earlier in another study with colitis [28].

### Statistical analysis

Data analysis was performed using IBM SPSS version 26. Data were expressed as mean ± SEM. The normality of the data was checked by skewness and kurtosis Z-values and the Shapiro-Wilk test. Data with normal distribution were analysed by one-way ANOVA with appropriate post hoc tests (based on homogeneity of variance) for pairwise intergroup comparisons. The effect size (partial eta squared) was also estimated. Non-parametric data were analysed by the Kruskal-Wallis H test for multiple groups, followed by posthoc pairwise comparisons. Statistical significance was fixed at a value of < 0.05.

## Results

### Isolation and structural identification of paradol from *A. melegueta*

The chloroform-soluble fraction of *A. melegueta* seeds was subjected to different chromatographic procedures to obtain 6-paradol. The identity of the isolated compound was confirmed through comparison with the previous literature [20] using their 1H and 13C NMR data (Supplementary data).

### Biochemical determination of colon inflammation

As depicted in Table 1 and Table 2 (based on distribution of data), there was a significant decrease in colonic GSH and significant increases in colonic MPO and colonic MDA levels in the acetic acid-induced colitis group compared to the normal control. Additionally, all the groups differed significantly in measurements of colonic GSH, colonic MPO, and colonic MDA (*p* < 0.001) compared to the untreated acetic acid-induced colitis group. Furthermore, post hoc intergroup comparison tests analysing colonic GSH revealed no significant differences amongst the normal control, sulfasalazine (500 mg/kg) and 6-paradol (200 mg/kg) groups, meaning that a high dose of 6-paradol significantly reversed the changes in colonic GSH. Regarding colonic MPO and MDA, pairwise comparison revealed that all treatment groups except low-dose 6-paradol (50 mg/kg) significantly reversed the changes in these parameters compared to the untreated colitis group. However, they did not reach levels comparable to the normal control, except for the high-dose 6-paradol (200 mg/kg) and sulfasalazine (500 mg/kg) groups. Interestingly, though the medium dose of 6-paradol (100 mg/kg) did not rectify the levels of colonic biochemical parameters to a statistically significant point per se, compared to the normal control or sulfasalazine-treated group, the effect size was fairly large (compared to the low-dose group), implying a reasonably good outcome from a therapeutic standpoint. This was further reinforced by non-significant differences with the high-dose 6-paradol group (200 mg/kg) in some of the parameters.

**Table 1:** Effect of 6-paradol on colon GSH, Serum GSH and Serum TNF-α, in acetic acid-induced colitis rats

Parameter	Group [Mean ± SEM (95%CI)]						F	***p***	Partial Eta Squared (η^**2**^_**p**_)
	NC	AC	SG	LT	MT	HT			
Colon GSH^a,b^ (mmol/mg)	6.71 ± 0.32 (5.95, 7.47)	1.92 ± 0.08 (1.73, 2.11)	6.23 ± 0.15 (5.86, 6.61)	3.82 ± 0.13 (3.51, 4.13)	4.47 ± 0.17 (4.06, 4.88)	6.45 ± 0.25 (5.84, 7.05)	85.27	0.001	0.900
Serum GSH^a,c^ (μmol/l)	12.82 ± 0.54 (11.54, 14.10)	4.10 ± 0.10 (3.85, 4.34)	11.20 ± 0.37 (10.32, 12.07)	5.96 ± 0.18 (5.52, 6.40)	8.73 ± 0.33 (7.94, 9.52)	10.97 ± 0.20 (10.49, 11.45)	108.35	0.001	0.919
Serum TNF-α^a,b^ (pg/ml)	18.41 ± 0.09 (16.27, 20.55)	51.25 ± 1.50 (47.68, 54.81)	19.11 ± 0.84 (17.10, 21.12)	41.53 ± 2.26 (36.19, 46.88)	30.05 ± 0.51 (28.82, 31.27)	22.62 ± 0.55 (21.31, 23.93)	112.38	0.001	0.922

Legend: Effects of 6-paradol at different doses on colon GSH, serum GSH and serum TNF-α in acetic acid-induced colitis rats, analysed by one-way ANOVA followed by a posthoc Tukey’s test for pairwise comparison; ^a^significant difference with AC (All parameters); ^b^significant difference with NC (for colonic GSH, and Serum TNF-α); ^c^significant difference with NC (for serum GSH); Effect size (η2p); *NC* Normal control, *AC* Acetic acid-induced colitis untreated, *SG* Sulfasalazine treated (500 mg/kg), *LT* Low-dose 6-paradol (50 mg/kg), *MT* Medium-dose 6-paradol (100 mg/kg), *HT* High-dose 6-paradol (200 mg/kg); *GSH* Glutathione, *TNF* Tumour necrosis factor

**Table 2:** Effect of 6-paradol on Colon MPO and MDA, Serum MDA and IL-6, in acetic acid- induced colitis rats

Parameter	Group [Mean ± SEM (95%CI)]; Mean Rank (***Italics***)						Kruskal-Wallis H	***p***
	Normal Control (NC)	Acetic acid induced colitis: Untreated (AC)	Sulfasalazine treated (500 mg/kg) (SG)	6-paradol (50 mg/kg) (LT)	6-paradol (100 mg/kg) (MT)	6-paradol (200 mg/kg) (HT)		
Colonic MPO^a,b^ (unit/g)	12.28 ± 0.44 (11.24, 13.32)	49.80 ± 1.61 (45.97, 53.62)	16.020 ± 0.66 (14.61, 17.78)	41.56 ± 1.14 (38.85, 44.26)	26.26 ± 0.88 (24.16, 28.35)	17.58 ± 0.75 (15.80, 19.37)	44.35	0.001
	*4.50*	*44.00*	*14.81*	*37.00*	*28.50*	*18.19*		
Colonic MDA^a,b^ (mmol/mg)	16.90 ± 0.49 (15.73, 18.06)	44.20 ± 1.68 (40.20, 48.19)	17.57 ± 0.77 (15.74, 19.40)	36.17 ± 0.81 (34.25, 38.09)	25.57 ± 0.88 (23.49, 27.65)	18.27 ± 0.98 (15.95, 20.59)	40.46	0.001
	*10.56*	*44.13*	*12.81*	*36.88*	*28.44*	*14.19*		
Serum IL-6^a,b,c^ (pg/ml)	12.01 ± 0.31 (11.25, 12.74)	41.53 ± 1.11 (38.90, 44.16)	12.93 ± 0.40 (11.98, 13.89)	35.57 ± 1.54 (31.92, 39.22)	25.86 ± 0.74 (24.11, 27.61)	14.71 ± 0.78 (12.85, 16.56)	42.30	0.001
	*7.19*	*43.75*	*12.25*	*37.00*	*28.75*	*18.06*		
Serum MDA^a,b,c^ (nmol/l)	21.96 ± 0.75 (20.17, 23.75)	84.35 ± 2.03 (79.53, 89.16)	22.27 ± 0.66 (20.70, 23.84)	68.61 ± 1.76 (64.43, 72.78)	47.50 ± 2.29 (42.07, 52.92)	26.61 ± 0.85 (24.59, 28.62)	43.23	0.001
	*9.31*	*44.25*	*8.81*	*36.75*	*28.50*	*19.38*		

Legend: Effects of 6-paradol in different doses on colon MPO, colon MDA, serum MDA, and serum IL-6 in acetic acid-induced colitis rats, analysed by the Kruskal-Wallis H test followed by pairwise comparison; ^a^significant difference with AC (All parameters). ^b^significant difference with NC (All parameters); ^c^LT vs MT; MT vs HT for serum IL-6 and serum MDA; *NC* Normal control, *AC* Acetic acid-induced colitis untreated, *SG* Sulfasalazine treated (500 mg/kg), *LT* Low-dose 6-paradol (50 mg/kg), *MT* Medium-dose 6-paradol (100 mg/kg), *HT* High-dose 6-paradol (200 mg/kg), *MPO* Myeloperoxidase, *MDA* Malondialdehyde, *IL* Interleukin

### Determination of inflammatory markers and antioxidants in serum

Like colonic parameters, serum parameters also revealed similar patterns (Tables 1 and 2). Serum GSH levels decreased, while serum TNF-α, IL-6, and MDA increased significantly in the acetic acid-induced colitis group compared to the normal control. Additionally, all groups differed significantly in serum GSH and serum TNF-α compared to the untreated acetic acid-induced colitis group. Intergroup pairwise comparison revealed non-significant differences amongst the normal control, sulfasalazine (500 mg/kg), and 6-paradol (200 mg/kg) groups in serum TNF-α, indicating a near normalization of serum TNF-α by sulfasalazine and high dose 6-paradol (200 mg/kg). For serum GSH, neither sulfasalazine (500 mg/kg) nor any of the treatment groups were able to reach levels comparable to the normal control. However, no significant difference was observed between the high-dose 6-paradol (200 mg/kg) and sulfasalazine (500 mg/kg) groups (*p* = 0.998), suggesting sufficient protection. Furthermore, a large effect size (91.9%) between different treatment groups further indicates a fair therapeutic response. For serum MDA and serum IL-6, pairwise analysis revealed that all treatment groups [except low-dose 6-paradol (50 mg/kg)] significantly reversed the changes in these parameters compared to the untreated acetic acid-induced colitis group (*p* < 0.001), but they did not reach levels comparable to the normal control except for the high-dose 6-paradol (200 mg/kg) and sulfasalazine (500 mg/kg) groups. In addition, non-significant differences were identified between the low- and medium-dose and between the and medium- and high-dose 6-paradol groups, indicating a dose-dependent response.

### Macroscopic assessment of colonic damage

**Fig. 1:**
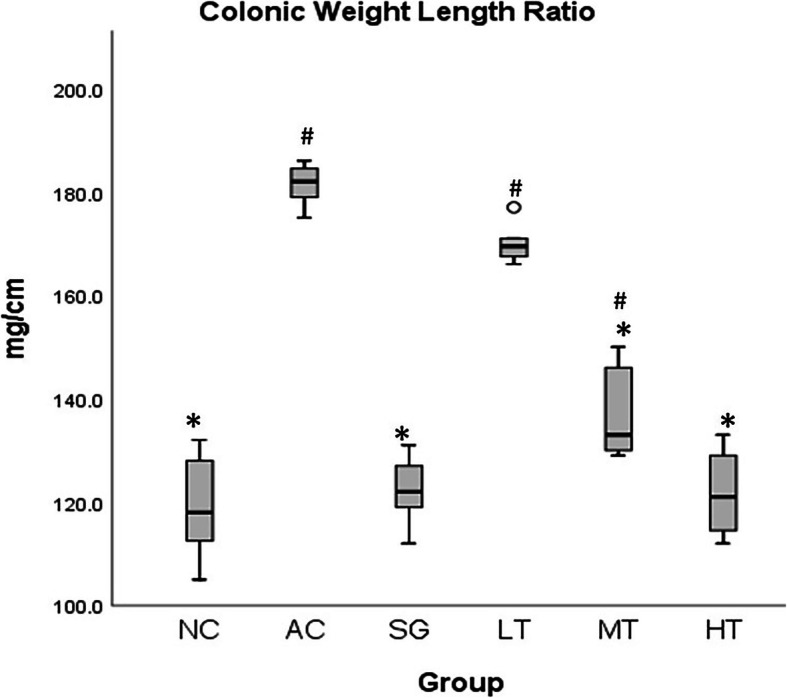
Effect of 6-paradol on the colon weight length ratio in acetic-induced acid colitis in rats. Legend: Effect of 6-paradol in different doses on the colon weight-length ratio in acetic acid-induced colitis rats analysed by the Kruskal-Wallis H test followed by pairwise comparison. Data are expressed as Mean ± SEM. NC=Normal control, AC = Acetic acid-induced colitis untreated, SG = Sulfasalazine treated 500 mg/kg, LT = Low-dose 6-paradol (50 mg/kg), MT = Medium-dose 6-paradol (100 mg/kg), HT = High-dose 6-paradol (200 mg/kg); *significant vs. AC: All groups except LT; #significant vs. NC: AC, LT, and MT; Kruskal-Wallis H = 37.75, *p* < 0.001

As depicted in Fig. 1, there was a significant increase in the colonic weight to length ratio in acetic acid-induced colitis rats compared to the normal control. All treatments except low-dose 6-paradol (50 mg/kg) significantly reversed the changes in these parameters. In the high-dose 6-paradol (200 mg/kg) and sulfasalazine (500 mg/kg) groups, the colonic weight to length ratio almost normalized and reached levels similar to the normal control. It is also worth noting that though the medium-dose 6-paradol group (100 mg/kg) did not reduce the ratio to statistical significance compared to the normal control, this reduction was not unimportant. This finding was further supported by a non-significant difference with the high-dose 6-paradol (200 mg/kg) and sulfasalazine (500 mg/kg) treated groups (*p* = 0.081). Moreover, macroscopic evidence also supported this conclusion. The colon showed severe colonic lesions, such as o such as edema, haemorrhages, necrosis, and ulceration, in the acetic acid-induced colitis group. 6-Paradol offered dose-dependent protection against these changes, and the colon in the high-dose 6-paradol group looked as good as the colon in the sulfasalazine-treated group.

### Histopathology

**Fig. 2:**
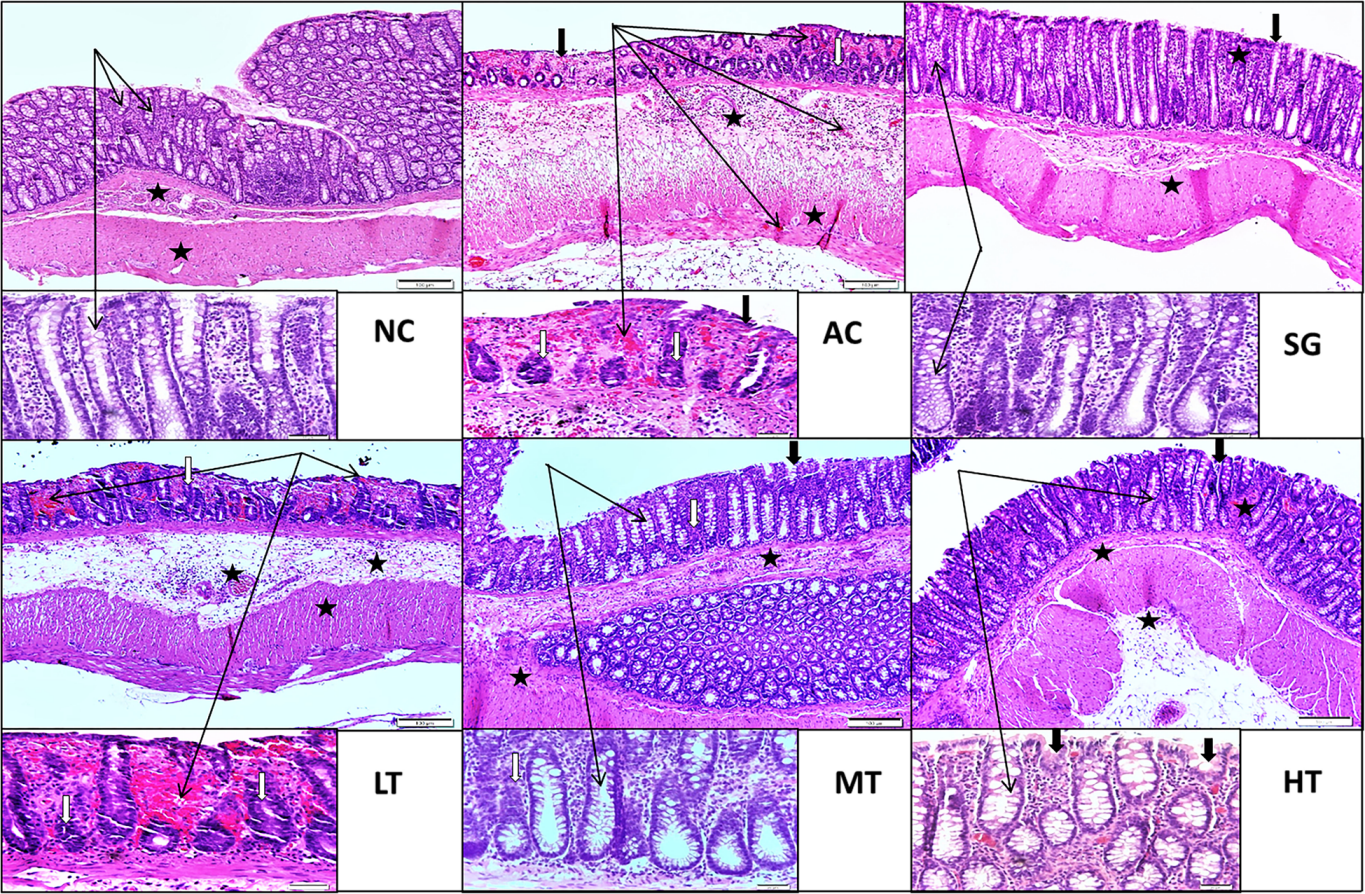
Effect of 6-paradol on H&E staining of acetic acid-induced colitis rat colon. Legend: Effect of 6-paradol at different doses on H&E staining of acetic acid-induced colitis rat colon. NC=Normal control, AC = Acetic acid-induced colitis untreated, SG = Sulfasalazine treated 500 mg/kg, LT = Low-dose 6-paradol (50 mg/kg), MT = Medium-dose 6-paradol (100 mg/kg), HT = High-dose 6-paradol (200 mg/kg). The normal control group (NC) shows well-preserved mucosal crypts, no hypertrophy of goblet cells, normal appearing mucosal lining (black arrows), and healthy submucosa with no inflammation (star). The AC group shows severe active colitis with mucosal erosions, ulceration, and mucosal atrophy (thick black arrows). Haemorrhage is also evident in the mucosa and submucosa (black arrows). There is crypt destruction, dilatation, reduced goblet cells (white arrows), and marked transmural inflammation, including the mucosa and submucosa (star). The SG group shows an almost preserved mucosa with trivial inflammation (thick black arrow), with slightly dilated but overall healthy crypts showing insignificant goblet cell hyperplasia and mucus (black arrows). A mild polymorphic infiltration in otherwise healthy mucosa and submucosa can also be seen (star). The LT group shows mucosal erosions, ulcerations, destruction of mucosal lining, inflammation and haemorrhagic foci (black arrows). Obliteration and destruction of crypts is also manifested (white arrows). Haemorrhagic foci in the submucosa, in addition to inflammatory infiltration, can also be appreciated (star). The MT group shows minor inflammation of the mucosal lining (thick black arrow). However, the cryptic structures are relatively preserved, with only mild dilatation (black arrows). Nevertheless, a number of crypts are infiltrated with inflammatory cells (cryptitis) (white arrow). Some inflammation is also present in the submucosa (star). The HT group shows largely preserved mucosal lining with insignificant inflammation (thick black arrows) and almost undamaged, non-dilated, non-necrotic crypts (black arrows). Nevertheless, there are some prominent goblet cells and foci of micro-haemorrhages, but submucosa is largely unremarkable (star)

As depicted in Fig. 2, acetic acid-induced colitis rats showed erosion of surface epithelium, severe ulceration, mucosal atrophy, mucosal and submucosal haemorrhages and inflammatory cell infiltration. Crypt destruction and dilatation also occurred. These changes were partially reversed in the 6-paradol (100 mg/kg) group and were substantially reversed in the 6-paradol (200 mg/kg) and sulfasalazine (500 mg/kg) groups. The moderate-dose 6-paradol group (100 mg/kg) exhibited minor inflammation of the mucosal lining, with relative preservation, and mildly dilated cryptic structures, but with mild to moderate cryptitis. Treatment with high-dose 6-paradol (200 mg/kg) revealed a largely preserved mucosal lining with insignificant inflammation, non-dilated, non-necrotic crypts, albeit with some haemorrhagic foci, but the sub-mucosa was largely unremarkable.

## Discussion

Ulcerative colitis is described as an inflammatory and relapsing chronic gut disorder that possibly occurs from interactions of genetic, environmental, dietary, and pro-inflammatory factors, alongside altered immune responses, oxidative stress, and gut microbiome dysfunction. Chemotaxis and inflammatory cell infiltration and the release of mediators and proteases occur, eventually resulting in ulceration, haemorrhage, necrosis, and intestinal tissue damage [29, 30]. Acetic acid-induced colitis is a well-investigated method for creating an animal model of UC, as it represents a pathology similar to UC in humans that consists of an increase in inflammatory mediators, localized involvement and damage to the intestinal epithelium [22].

The colitis untreated group in our study presented with significant elevation of colonic MPO activity, which indicates neutrophil infiltration and inflammatory cascade dysfunction, documented not only in animal models but also in IBD patients [31, 32]. Colonic and serum MDA levels were also elevated, and colonic and serum GSH levels were reduced significantly, suggesting an increase in oxidative stress-mediated injury in accordance with previous studies [29, 33]. Serum IL-6 was also elevated in acetic acid-induced colitis rats in our study. This elevation has been correlated with disease severity in UC patients and has been documented to play crucial roles in pro-inflammatory processes and the dysregulation of immune function [34]. As mentioned hitherto, TNF-α is a key mediator in pathogenesis of UC and is upregulated in many patients [35], causing neutrophil infiltration through increased levels of adhesion molecules, producing tissue damage [36].

We also found profound mucosal ulceration, transmural inflammation, haemorrhage, oedema, crypt damage, and necrosis in colitis-induced untreated rats. Our findings are in accordance with previous studies investigating therapeutic options for IBD [37, 38]. It has also been reported that sulfasalazine (500 mg/kg) pretreatment substantially mitigated the biochemical and microscopic changes of acetic acid-induced colitis [39]. Our study showed that 6-paradol, especially at a dose of 200 mg/kg, significantly reversed the biochemical changes induced by colitis. The improvements were as good as the changes in sulfasalazine-treated or normal rats. Macroscopic and histopathological findings also revealed a dose-dependent reversal, with the highest protection at 6-paradol 200 mg/kg, similar to sulfasalazine or normal rats, moderate protection at 6-paradol 100 mg/kg, and mild or no protection at 6-paradol 50 mg/kg, which was similar to colitis-untreated rats.

6-Paradol (IUPAC name [1-(4-hydroxy-3-methoxyphenyl) decan-3-one]) is a pungent phenolic compound found in plants of the Zingiberaceae family, such as ginger and grains of paradise (*Aframomum melegueta* or alligator pepper). As mentioned elsewhere, a few studies have reported the antioxidant, anti-inflammatory, cytotoxic, anti-hyperlipidaemic, hypoglycaemic, and antitumour activities of different paradol compounds in various animal models, but to the best of our knowledge, no study has yet reported the protective effect in a colitis model in rats.

Chung et al. 2001, demonstrated that 6-paradol attenuated hydrogen peroxide production, MPO activity and other oxidant enzymes in mouse skin [40]. Anti-inflammatory activity has also been demonstrated by 6-paradol and 6-gingerol through a reduction in TNF-α production in female mice [41] and through the inhibition of cyclo-oxygenase-2 enzyme [42]. A study in a neuro-inflammatory model also reported significant attenuation of TNF-α and IL-6 by 6-paradol [20]. As TNF-α is a Th1 cytokine, and IL-6 is a Th2 cytokine, reduction of these parameters in our study by 6-paradol suggests anti-inflammatory activity through modulation of T-cell immune responses [43]. The same study also reported that 6-paradol decreased nitric oxide (NO) production through inhibiting the upregulation of iNOS. It is being increasingly documented that NO overproduction by iNOS upregulation is associated with IBD (especially UC). Hence, 6-paradol carries an additional benefit in UC by checking unabated NO production [44].

Mucosal injury has a direct relationship with glutathione levels [45], and its replenishment by 6-paradol correlates with the mucosal protection seen in our study. This is reinforced by a report from Suresh et al. 2010, reporting normalization of GSH levels by 6-paradol [46]. Likewise, El-Halawany et al. 2014 also reported a reduction in TNF-α and GSH in hepatocyte injury by 6-paradol [47].

In addition to the beneficial effects discussed above, 6-paradol also inhibits α-glucosidase in the intestine [48]. It has been documented that the inhibition of this enzyme by the anti-diabetic drug acarbose induces H_2_, which causes reductions in the levels of oxidative stress markers, such as MPO, thus improving the symptoms in ulcerative colitis [49]. Hence, this might be an additional beneficial action of 6-paradol in UC.

Moreover, various actions of sulfasalazine in IBD, such as the inhibition of macrophage-derived cytokines (TNF-α and IL-6), the increased expression of PPAR-γ, the inhibition of COX-2, the activation of caspase 3, and the suppression of NF-*κ*B activation, are also reported from 6-paradol [50–52]. Hence, from the discussion above, it can be conferred that 6-paradol from *A. melegueta* seeds plays key roles in inflammation, immunomodulation, cellular oxidation, proliferation, and repair mechanisms, and these process are implicated in the pathophysiology of inflammatory bowel disease.

The limitation of the study is the sample size and the small number of outcome variables measured at the end. For example, IL-6 and TNF-α should have been evaluated at the site of the lesion on gut tissue. Criteria for evaluation of ulcerative colitis, such as ‘disease activity index’, microscopic scoring, and spleen weight, should also have been included. Moreover, the pathophysiology of ulcerative colitis in humans is complex and includes an interplay of dietary, genetic and environmental factors, which are extremely difficult to replicate in an animal experimental study.

## Conclusion

The present study concludes that 6-paradol, an active constituent from the seeds of *A. melegueta,* is effective against acetic acid-induced colitis in rats, probably by anti-inflammatory, antioxidant, and mucosal protective actions. To our best knowledge, this effect is being reported for the first time in a colitis model, proposing a possible benefit in the management of IBD. These findings encourage the scientific community to pursue further research in this domain.

## Supplementary Information

**Additional file 1.**

## Data Availability

The datasets used and/or analysed during the current study are available from the corresponding author on reasonable request.

## Abbreviations

GSH: Glutathione
MDA: Malondialdehyde
MPO: Myeloperoxidase
TNF-α: Tumour Necrosis Factor alpha
IL-6: Interleukin 6
CD: Crohn’s disease
UC: Ulcerative colitis
IBD: Inflammatory Bowel Disease
NO: Nitric Oxide
COX: Cyclooxygenase
