# Correction to: Protective effect of 6-paradol in acetic acid-induced ulcerative colitis in rats

**DOI:** 10.1186/s12906-021-03241-1

**Published:** 2021-02-10

**Authors:** Misbahuddin Rafeeq, Hussam Aly Sayed Murad, Hossam Mohammed Abdallah, Ali M. El-Halawany

**Affiliations:** 1 Department of Pharmacology, Faculty of Medicine, King Abdulaziz University (KAU), Rabigh Campus, Jeddah, 21,589 Saudi Arabia; 2 Department of Pharmacology, Faculty of Medicine, Ain Shams University, Cairo, 11,562 Egypt; 3 Department of Natural Products and Alternative Medicine, Faculty of Pharmacy, KAU, Jeddah, 21,589 Saudi Arabia; 4 Department of Pharmacognosy, Faculty of Pharmacy, Cairo University, Cairo, 11,562 Egypt

**Correction to: BMC Complement Med Ther 21, 28 (2021)**

**https://doi.org/10.1186/s12906-021-03203-7**

Following publication of the original article [1], the authors reported an error in affiliation of the last co-author, Ali M. El-Halawany should have both affiliations 3 and 4.

The updated author affiliations should be:

Misbahuddin Rafeeq^1^*, Hussam Aly Sayed Murad^1,2^, Hossam Mohammed Abdallah^3,4^ and Ali M. El-Halawany^3,4^

1 Department of Pharmacology, Faculty of Medicine, King Abdulaziz University (KAU), Rabigh Campus, Jeddah 21,589, Saudi Arabia.

2 Department of Pharmacology, Faculty of Medicine, Ain Shams University, Cairo 11,562, Egypt.

3 Department of Natural Products and Alternative Medicine, Faculty of Pharmacy, KAU, Jeddah 21,589, Saudi Arabia.

4 Department of Pharmacognosy, Faculty of Pharmacy, Cairo University, Cairo 11,562, Egypt.

The original article [1] has been updated.

## References

[1] Rafeeq M, Murad HAS, Abdallah HM (2021). Protective effect of 6-paradol in acetic acid-induced ulcerative colitis in rats. BMC Complement Med Ther.

