# Supplementary figures and images for: Protective effect of 6-paradol in acetic acid-induced ulcerative colitis in rats
Source: BMC Complement Med Ther. 2021 Jan 13;21:28. doi: 10.1186/s12906-021-03203-7 (PMC7805070; doi:10.1186/s12906-021-03203-7)

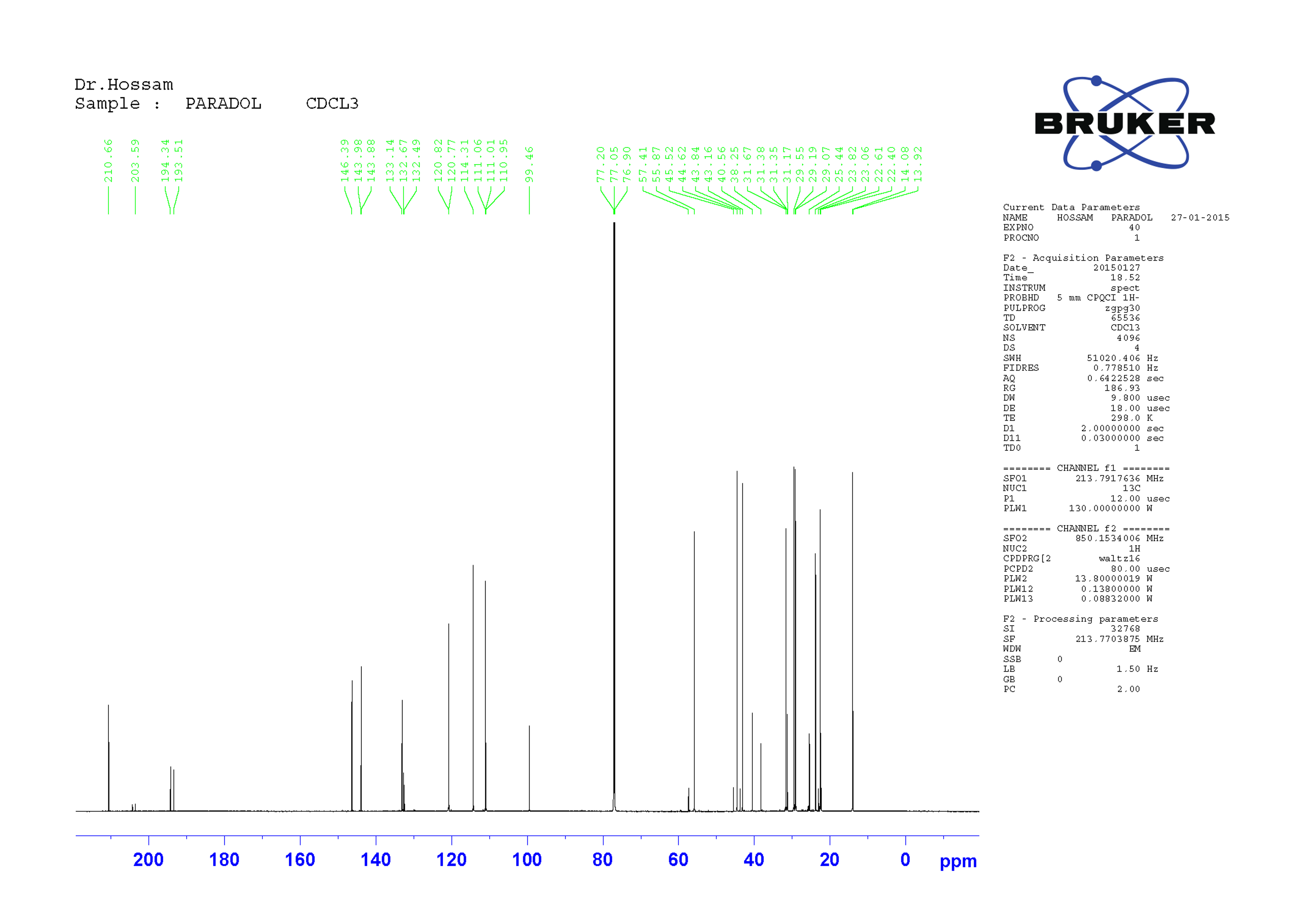

Supplement: Supplementary file 1 — Additional file 1. [file 12906_2021_3203_MOESM1_ESM.zip › PARADOL C-13.tiff]

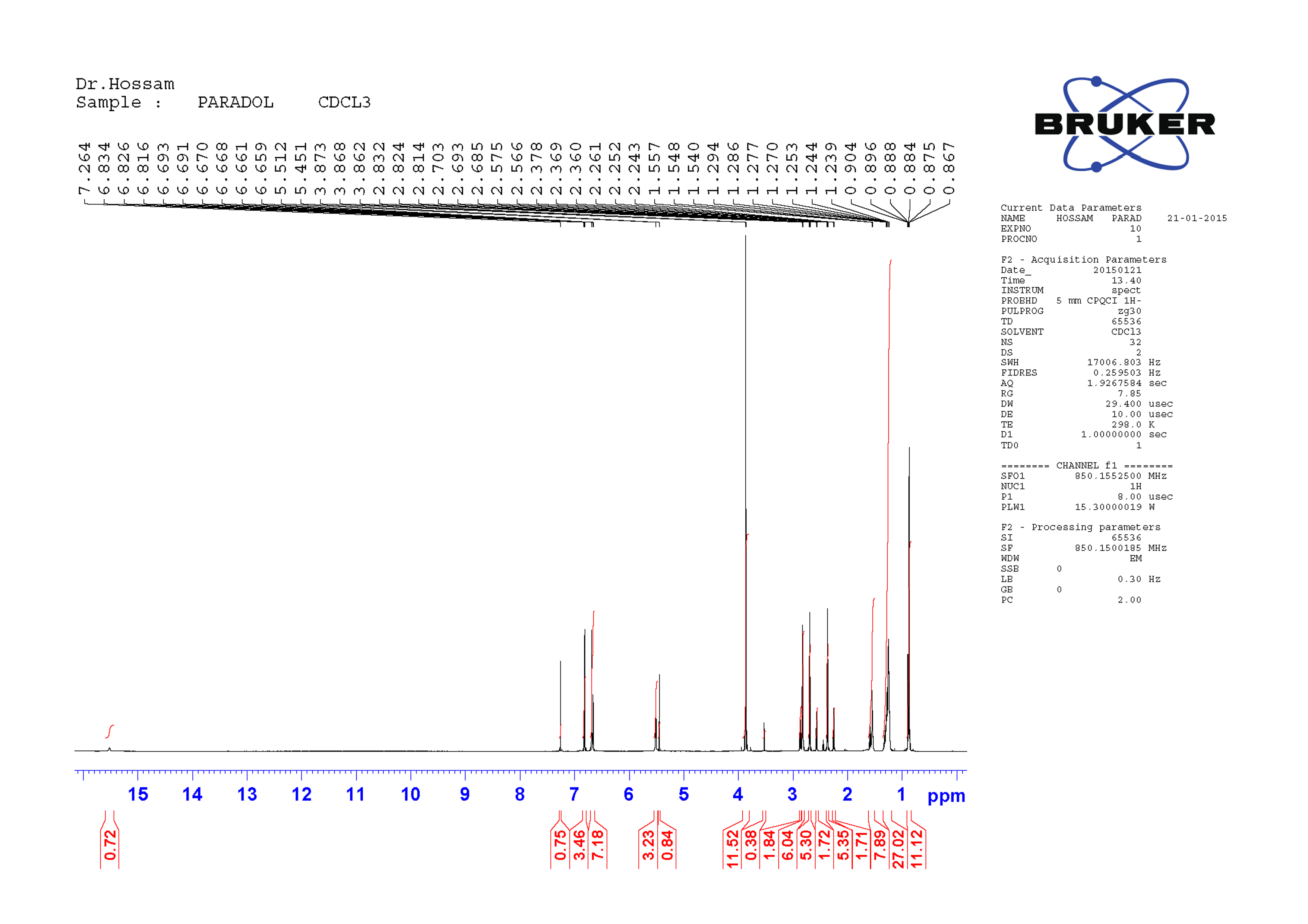

Supplement: Supplementary file 1 — Additional file 1. [file 12906_2021_3203_MOESM1_ESM.zip › PARADOL.tiff]
